# Calpain 1 gene expression in liver tissue and the association of novel calpain 1 single-nucleotide polymorphisms (SNPs) with meat quality in Bali cattle

**DOI:** 10.5194/aab-67-311-2024

**Published:** 2024-07-03

**Authors:** Dairoh, Mokhamad Fakhrul Ulum, Jakaria, Cece Sumantri

**Affiliations:** 1 Graduate School of Animal Production and Technology, Faculty of Animal Science, IPB University, Bogor 16680, Indonesia; 2 Department of Animal Production and Technology, Faculty of Animal Science, IPB University, Bogor 16680, Indonesia; 3 Division of Reproduction and Obstetrics, School of Veterinary Medicine and Biomedical Sciences, IPB University, Bogor 16680, Indonesia

## Abstract

Calpain 1 (*CAPN1*) is an enzyme that influences meat tenderization, and it is involved in post mortem proteolysis. The bovine *CAPN1* gene was chosen as a candidate gene for DNA sequencing to identify novel single-nucleotide polymorphisms (SNPs) in exons 8–10 and assess their associations with meat quality in Bali cattle. In an analysis of 95 Bali cattle, two novel SNPs (g.5327C > T and g.5959C > T) were identified in exons and four novel SNPs (g.5534C > T, g.5807A > C, g.5857G > A, and g.5869T > C) were discovered in introns. Allelic frequency was evaluated, and Hardy–Weinberg equilibrium was found for all SNPs in Bali cattle. The marbling score and intramuscular fat content as determined by ultrasound were associated with g.5869T > C and g.5959C > T. g.5327C > T was associated with pH and meat color in Bali cattle, whereas two other SNPs (g.5869T > C and g.5959C > T) were associated with Warner–Bratzler shear force and meat color. Furthermore, g.5869T > C was associated with linolenic acid content, and g.5959C > T with caprylic and linolenic acid levels. However, intronic SNPs (g.5534C > T, g.5807A > C, and g.5857G > A) did not significantly affect meat quality in Bali cattle. Quantitative real-time PCR of liver tissue revealed that the mRNA expression of *CAPN1* significantly differed ( P<0.05 ) among the CT, CC, and TT genotypes. The results suggest that genetic variability in loci within *CAPN1* might be associated with meat quality in Bali cattle.

## 1 Introduction

The market for high-yielding beef cattle breeds has been affected by the recent large growth in the demand for beef, which is a crucial source of protein (Smith et al., 2018). Bali cattle is native Indonesian domesticated cattle (Martojo, 2012) that is used to produce good-quality meat. Bali cattle exhibit an extremely close kinship with the banteng (*Bibos banteng*), but they are distantly related to *Bos taurus* and *Bos indicus* based on an analysis of the D-loop mtDNA gene (Jakaria et al., 2019). The meat from Bali cattle is prized for its delicious taste and desirable texture. Consequently, these cattle are favored both by local farmers and consumers who prioritize high-quality sustainable meat production. Bali cattle have a compact body conformation with a carcass presentation rate of up to 56 % (Hafid et al., 2019). Bali cattle can attain United States Department of Agriculture (USDA) quality standards for beef at a relatively young age, namely 1.5–2.5 years, highlighting their efficiency in meat production (Aditia et al., 2014). High production of class I back cuts, especially cube roll and strip loin, can be achieved using Bali cattle (Aditia et al., 2014).

The meat quality of Bali cattle has been improved through enhanced management practices. One such strategy is crossbreeding Bali cattle with other cattle, including Wagyu cattle, to obtain combinations of beneficial genes to increase livestock productivity and generate a new breed for long-term production. Although crosses between Wagyu and Angus have been achieved (Liu et al., 2021), no crossbreeding between Wagyu and Bali has been reported.

Meat quality has a significant impact on crucial aspects of consumer acceptance, including the level of satisfaction among consumers, producers, and the meat industry at large (Khasrad et al., 2017). Meat quality indicators that significantly influence consumer acceptance include flavor, tenderness, marbling, and color, all of which have an important economic value (Geletu et al., 2021). Therefore, improving the quality of Bali beef is essential for supporting food security in Indonesia and ensuring the availability of high-quality meat that can meet the needs of the population. According to Berry and Kearney (2018), environmental factors such as feed quality, age at slaughter, management practices, and processing at the time of slaughter affect meet quality and genetic factors also have a significant influence on meat quality. Many genes affect the quantitative characteristics of meat. Selection based on phenotypic data is considered less efficient because environmental influences are high. Therefore, the marker-assisted selection method exhibits excellent potential because of its time and cost efficiencies and high accuracy (Nayak and Singh, 2017). In marker-assisted selection, molecular markers are used to map and identify genetic loci associated with traits of interest, thereby enabling more precise and focused genetic selection. Screening potential parents based on genetic information can help minimize environmental influences, increase selection precision, and accelerate genetic progress (Goddard, 2009).

In recent years, several genetic markers associated with variations in beef quality have been identified. Genome-wide association studies have been conducted to identify putative genes for meat quality. The proteolytic calpain system consists of the genes calpain 1 (*CAPN1*) and calpastatin (*CAST*), both of which required a high calcium concentration to function. Meat quality traits, particularly tenderness, are associated with *CAPN1* (Mateescu et al., 2017). Myofibril proteins are degraded by calpain through post mortem proteolysis (Coria et al., 2018). Certain *CAST* enzymes suppress the proteolysis of calpain, making it inactive when calcium is present. Based on research conducted by Dairoh et al. (2021), the *CAPN1* gene has been consistently demonstrated to affect the meat quality of Bali cattle. In their study, six single-nucleotide polymorphisms (SNPs) and eight indels in the *CAPN1* gene were found to significantly affect variables related to meat quality, and these genetic variations were not found in *B. indicus* or *B. taurus* (Dairoh et al., 2022).

This study investigated the effects of SNPs within the *CAPN1* gene on meat quality in Bali cattle. Through an analysis of SNP genetic diversity and an examination of the associations of SNPs with meat quality traits, specific genotypes influencing these aspects were identified. The second aim was to determine the association of *CAPN1* gene expression with tenderness and fatty acid composition in Bali cattle. The findings of this research hold significant implications for the advancement and selection of Bali cattle. These findings will contribute to progress in genetic breeding practices aimed at enhancing meat quality within the Bali cattle population.

## 2 Material and methods

### 2.1 Blood sampling

Blood samples from 95 Bali cattle were collected in Kupang, East Nusa Tenggara province, Indonesia, and shipped to a Basirih slaughterhouse. The cattle were slaughtered at the Basirih slaughterhouse in south Banjarmasin, South Kalimantan province. Venoject was used to collect blood samples from the jugular vein of each animal, and samples were stored in a 1.5 mL EDTA vacuum container. This experimental procedure was approved by the Animal Ethics Committee of the Banjarmasin City Food Security, Agriculture, and Fisheries Service (approval no. 520/624/DKP3/X11/2021). DNA extraction was performed at the Animal Molecular Genetics Laboratory, Faculty of Animal Husbandry, IPB University. The DNA extraction method using the Geneaid kit was used for extraction.

### 2.2 Marbling score and intramuscular fat measurement

**Figure 1:**
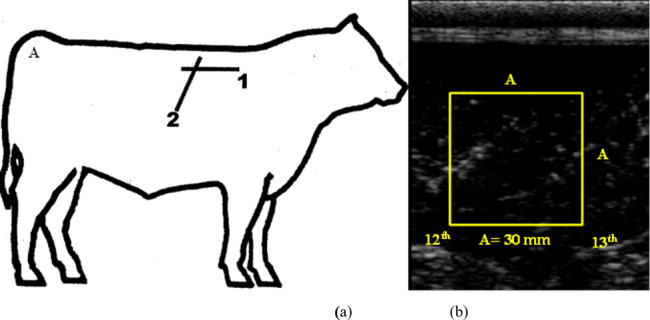
The position of the muscle ultrasound measurement at the 12th–13th rib (1: longitudinal; 2: transversal) **(a)** and image analysis using ImageJ software **(b)**. (A: measurement area, 30mm×30mm .)

A 7.1 MHz ultrasound transducer was used to measure the marbling score and intramuscular fat content at the 12th–13th rib position (Ulum et al., 2014; BIF, 2016). Sonograms of intramuscular fat were obtained 3 d before slaughter. To prepare for scanning, the skin of living Bali cattle was shaved. Ultrasound gel was used to allow ultrasound waves to pass from the probe to the tissue of the cattle. The images were immediately processed on the monitor screen, and a picture of the transversal intramuscular fat content was displayed. Ultrasound images were saved and analyzed using ImageJ software (NIH, Bethesda, MD, USA; Fig. 1). The Australian Meat Standard was used to determine the marbling score.

### 2.3 Physical analysis

In total, 250 g of meat was taken from the tenderloin of 44 Bali cattle. The assessed physical characteristics included pH, tenderness, cooking loss, color, and water-holding capacity (WHC). pH was analyzed using a calibrated pH meter. For tenderness and cooking loss analysis, 100 g of meat was boiled until the temperature reached 80 °C. Meat samples were treated with a 1.27 cm beef corer to make small cuts and had their Warner–Bratzler shear force (WBSF) measured. The difference in weight between fresh and cooked meat was used to calculate cooking loss. The meat color was analyzed using red litmus paper. The press method was used to determine WHC. For the analysis, Whatman filter paper was used. The wet area was the water area formed by the difference between the outer and inner circles absorbed by the paper because of the clamping method.

### 2.4 Fatty acid analysis

Fatty acid composition was determined using the Association of Official Analytical Chemists method (AOAC 2012). A chloroform–methanol solution was used to extract lipids from 40 g of meat. The recovered lipids were then transesterified to yield fatty acid methyl esters (FAMEs). The FAMEs were extracted with hexane solution, centrifuged, and dried. The resultant FAME products were dissolved in chloroform solution before being filtered to eliminate undesirable chemicals using solid-phase extraction. One microliter of FAMEs was placed in gas chromatography (GC) equipment. The isolated fatty acids were identified and quantified by mass spectrometry. The retention time and peak were recorded for each fatty acid component. Comparing the retention time to standards yields information on each fatty acid component. GC was used to identify saturated, monounsaturated, and polyunsaturated fatty acids.

### 2.5 Primer design and DNA amplification

*CAPN1* primer sequences were designed from exon 8 to exon 10 using the GenBank sequence of the National Center for Biotechnology Information (NCBI) (access code AH009246.3). The length of the primer sequences was determined using the Primer3 and Primer-BLAST websites, a Multiple Primary Analyzer, and Primary Stats software. The primer sequences were as follows: forward 5 ${}^{\prime }$ -GTG AAC TAC CAG GGC CAG AT-3 ${}^{\prime }$ and reverse 5 ${}^{\prime }$ -TCG TAC AGG GTG GTG TTC CA-3 ${}^{\prime }$ . The PCR product was 763 bp in length. The *CAPN1* gene was amplified using an AB System thermocycler and the following PCR conditions: predenaturation at 95 °C for 1 min; 35 cycles of denaturation at 95 °C for 15 s, annealing at 57 °C for 15 s, extension at 72 °C for 10 s; and final extension at 72 °C for 3 min. PCR products were separated by 1 % agarose gel in electrophoresis and examined using a UV Transilluminator (Bio-Rad, Hercules, CA, USA).

### 2.6 DNA sequencing and genotyping

The amplification product was sequenced on an ABI PRISM with the BigDye Terminator kit v3.1 by 1st Base Laboratory Services (Selangor, Malaysia). FinchTV, BioEdit, and Molecular Evolutionary Genetic Analysis (MEGA X) were used to analyze the sequencing results. The SNP positions were named after the complete NCBI reference sequence calculated from the first base of the *CAPN1* gene.

### 2.7 Extraction of total RNA and cDNA quantification

Total RNA was extracted from liver tissue using the RNeasy Fibrous Tissue Mini Kit based on the association (SNP g.5959C > T) with different meat tenderness and fatty acid composition phenotypes of Bali cattle. RNA was extracted from liver tissue as described by Khansefid et al. (2018). The liver is a central organ responsible for glycogen production and fatty acid metabolism. Meat tenderness is influenced by the inherent qualities of the meat, particularly the glycogen content. The liver is the primary organ for glucose production, contributing 85 %–90 % of the body's glucose supply and storing glycogen to regulate blood glucose levels (Bergman et al., 1974). The regulation of muscle glycogen levels affects the meat's ultimate pH, color, and tenderness through post mortem glycolytic changes (Rosenvold et al., 2001). Furthermore, fatty acid metabolism and synthesis are predominantly orchestrated within the liver, which is pivotal in lipid metabolism. This organ is responsible for synthesizing, catabolizing, and modifying fatty acids (Alves-Bezerra and Cohen, 2017). Various enzymes and proteins involved in fatty acid metabolism are synthesized in the liver, making it a crucial organ for studying the genetic regulation of these processes (Vallim and Salter, 2010). Therefore, it was reasonable to assess gene expression utilizing liver tissue when investigating tenderness and fatty acid composition phenotypes along with their associated processes.

**Table 1:** Primer information for the bovine *CAPN1* gene and a housekeeping gene, β -actin.

No.	Gene	Primer sequences	Amplified length (bp)
1	*CAPN1*	F: 5 ${}^{\prime }$ -TTTTCCGTGACGAGGCTTTC-3 ${}^{\prime }$ , F: 5 ${}^{\prime }$ -GAGGGTGTCATTGAGGGTAA-3 ${}^{\prime }$	227
2	β -actin	F: 5 ${}^{\prime }$ -ATGATATTGCTGCGCTCGTG-3 ${}^{\prime }$ , R: 5 ${}^{\prime }$ -GTGCTCAATGGGGTACTTGA-3 ${}^{\prime }$	212

*CAPN1*: calpain 1; F: forward; R: reverse.

Three groups based on the genotypes at the SNP g.5959C > T (CC, CT, and TT) were created to compare the relationships of *CAPN1* gene expression with tenderness and fatty acid levels. The t test was performed using SAS statistical software (SAS Institute, Cary, NC, USA) to measure the significance of differences among the three genotypes. Total RNA was treated using RNase-Free DNase (Promega, Madison, WI, USA), which was followed by RNA measurement using a spectrophotometer. The Transcriptor cDNA Synthesis Kit was used to convert RNA into cDNA. Using real-time PCR, cDNA was used to calculate *CAPN1* gene expression. Real-time PCR was performed with the SYBR Green master kit and a reaction mixture containing 5 µ L of the master kit, 0.5 µ L of the forward and reverse primers each (Table 1), 1 µ L of sample cDNA, and 3 µ L of NFW. Each cDNA sample and nontemplate control were examined in a 96-well microtiter plate in each run. The qRT-PCR machine was programmed to perform 5 min of predenaturation at 95 °C, 10 s of denaturation at 95 °C, 20 s of annealing at 54 °C, 30 s of extension at 72 °C, and 5 min of final extension at 72 °C. All samples were run in duplicate, and the target gene expression was normalized using the geometric mean of the housekeeping gene, β -actin. cDNA was quantified using the AG qTower Analytic Jena program (Listyarini et al., 2018).

**Figure 2:**
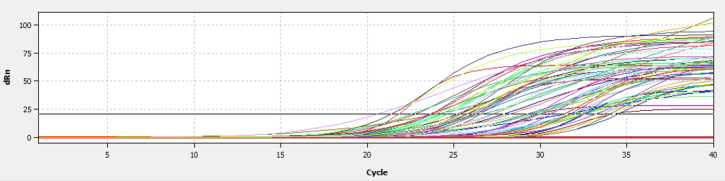
Construction of a fluorescence standard curve to calculate cycle threshold values by qRT-PCR.

**Figure 3:**
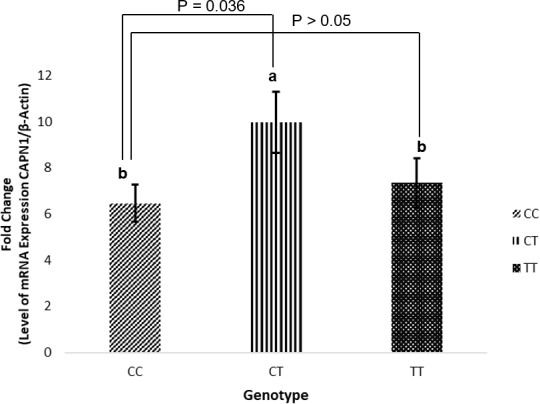
*CAPN1* mRNA expression in liver tissue based on genotype (CC, CT, and TT). mRNA expression is presented as the fold change using the β -actin gene as a reference. (*CAPN1*: calpain 1.)

*CAPN1* gene expression as determined by qRT-PCR was based on the absolute quantification method. This method utilizes a standard fluorescence curve to represent the number of cycles required for fluorescence to surpass the cycle threshold (Ct). The recorded Ct during the exponential amplification phase was used to calculate *CAPN1* gene expression (Fig. 2). This process involves comparing the sample CT to the standard curve, enabling the determination of the relative concentration of the *CAPN1* gene in the tested cDNA sample. Thus, the absolute quantification method allows for the direct and accurate measurement of gene expression in analyzed samples (Gamal and Ibrahim, 2024). The absolute quantification of gene expression by qRT-PCR was performed using Δ Ct expressed as a fold change, and the housekeeping gene β -actin was used as the reference gene. Δ Ct was calculated by subtracting the Ct of the target gene from that of the reference gene (Silver et al., 2006) as follows:

ΔCt=Cttarget-Cthousekeeping gene.

### 2.8 Data analysis

*CAPN1* gene diversity was investigated using the POPGENE 1.32 program, allele frequencies, genotype frequencies, observed and expected heterozygosity values, and the Hardy–Weinberg balance approach. The general linear model (GLM) method was used to investigate the relationships of *CAPN1* genotypes with the marbling score, intramuscular fat content, physical traits, and fatty acid levels. The mathematical formula for the GLM model is as follows:

Yij=μ+Gi+eij,

where Yij is the phenotypic observation, μ is the overall mean, Gi is the genotype effect, and eij is the random error.

**Table 2:** SNP information of the *CAPN1* gene.

Gene	SNP	Location	Variation type	SNP database	Amino acids
*CAPN1*	g.5327C > T	Exon 8	Transition	Novel	Asp/Asp
	g.5534C > T	Intron 8	Transition	Novel	–
	g.5807A > C	Intron 9	Transversion	Novel	–
	g.5857G > A	Intron 9	Transition	Novel	–
	g.5869T > C	Intron 9	Transition	Novel	–
	g.5959C > T	Exon 10	Transition	Novel	Asp/Asp

*CAPN1*: calpain 1; SNP: single-nucleotide polymorphism.

## 3 Results and discussion

### 3.1 Newly discovered SNPs in the *CAPN1* gene

Six SNPs were identified in the *CAPN1* gene using the chromatograms of double peaks from sequencing data. Two SNPs (g.5327C > T and g.5959C > T) were found in exons 8 and 10, respectively, whereas four SNPs (g.5534C > T, g.5807A > C, g.5857G > A, and g.5869T > C) were found in introns 8–9, as indicated in Table 2. Five SNPs (g.5327C > T, g.5534C > T, g.5857G > A, g.5869T > C, and g.5959C > T) in the *CAPN1* gene were transition mutations and one (g.5807A > C) was a transversion mutation. These SNPs were entirely synonymous, causing no amino acid changes. However, no studies have explored the influence of these polymorphisms on phenotypic traits. Direct DNA sequencing identified the six *CAPN1* SNPs as novel variations located between exons 8 and 10. The variants were was identified in the Bali sequence by aligning them with the entire ensemble (ENSBTAG00000010230) sequence of the cattle *CAPN1* gene using MEGA X software.

**Table 3:** Allelic and genotypic frequencies and diversity parameters of SNPs in the *CAPN1* gene.

SNP	N	Genotypic frequency				Allelic frequency		Ho	He	$\chi^{2}$ test
		AA	AB	BB		A	B			
g.5327C > T	95	0.64	0.32	0.04		0.80	0.20	0.316	0.322	0.033 ns
g.5534C > T	95	0.69	0.29	0.01		0.84	0.16	0.295	0.267	1.028 ns
g.5807A > C	95	0.64	0.34	0.02		0.81	0.19	0.337	0.309	0.804 ns
g.5857G > A	95	0.81	0.19	0.00		0.91	0.09	0.190	0.172	0.978 ns
g.5869T > C	95	0.08	0.42	0.49		0.29	0.71	0.421	0.418	0.005 ns
g.5959C > T	95	0.08	0.41	0.51		0.29	0.71	0.411	0.414	0.005 ns

*CAPN1*: calpain 1; SNP: single-nucleotide polymorphism; AA: reference genotype (wild-type); AB: heterozygous genotype; BB: mutant genotype; Ho: observed heterozygosity; He: expected heterozygosity; ns: not significant ( $\chi^{2}$ test) < chi-squared table (3.84: α0.05 df's 2).

### 3.2 *CAPN1* gene polymorphism

Table 3 presents the allelic frequencies, genotype frequencies, heterozygosity (expected heterozygosity, He, and observed heterozygosity, Ho), and Hardy–Weinberg equilibrium of the *CAPN1* gene. All SNPs in the *CAPN1* gene had three genotypes: AA, AB, and BB. All six discovered SNPs were polymorphic, with minor allele frequencies exceeding 5 % (Allendorf et al., 2013). According to the genetic diversity analysis, the AA genotype was the most common for g.5327C > T, g.5534C > T, g.5807A > C, and g.5857G > A, whereas the BB genotype was the most common for g.5869T > C and g.5959C > T. Heterozygosity can be used to estimate a population's genetic diversity. Ho was higher than He for g.5869T > C and g.5959C > T. This suggests the existence of high genetic heterogeneity at specific loci (Fang et al., 2012). All SNPs in the *CAPN1* gene were discovered to be in Hardy–Weinberg equilibrium. The Hardy–Weinberg equilibrium test is used to determine whether genotype proportions within a population remain constant throughout generations (Waples, 2015). The Bali cattle utilized in this investigation were obtained from breeders who used no selection and who did not deviate from the Hardy–Weinberg equilibrium (Garnier-Gere and Chikhi, 2013; Graffelman et al., 2017).

**Table 4:** Association of *CAPN1* gene SNPs with the marbling score and intramuscular fat percentage in Bali cattle.

SNP	Genotype ( n )	MS	IMF (%)
g.5327C > T	CC (58)	1.48±0.41	2.51±1.01
	CT (29)	1.54±0.76	2.66±1.89
	TT (4)	1.42±0.37	2.36±0.93
g.5534C > T	CC (63)	1.45±0.39	2.43±0.98
	CT (27)	1.62±0.78	2.85±1.95
	TT (1)	1.21± nc	1.83± nc
g.5807A > C	AA (58)	1.47±1.40	2.48±1.01
	AC (31)	1.56±0.74	2.72±1.85
	CC (2)	1.32±0.15	2.09±0.37
g.5857G > A	GG (73)	1.48±0.41	2.49±1.03
	GA (18)	1.60±0.89	2.80±2.23
g.5869T > C	TT (8)	$1.91\pm 1.28^{a}$	$3.56\pm 3.19^{a}$
	TC (39)	$1.41\pm 0.36^{b}$	$2.33\pm 0.89^{b}$
	CC (44)	$1.51\pm 0.44^{b}$	$1.56\pm 1.09^{b}$
g.5959C > T	CC (8)	$1.92\pm 1.27^{a}$	$3.59\pm 3.17^{a}$
	CT (88)	$1.38\pm 0.29^{b}$	$2.24\pm 0.74^{b}$
	TT (45)	$1.53\pm 0.47^{b}$	$2.62\pm 1.16^{b}$

*CAPN1*: calpain 1; MS: marbling score; IMF: intramuscular fat; nc: not counted. ${}^{a,b}$ Means in the same column with different superscripts were significantly different ( P<0.05 ). Values ​​with the superscript ${}^{a}$ indicate different and higher values compared to those with the superscript ${}^{b}$ .

### 3.3 Association of *CAPN1* gene SNPs with marbling and intramuscular fat

Table 4 displays the results of the associations of SNPs in the *CAPN1* gene with the intramuscular fat percentage and marbling score of Bali cattle. The phenotypes of intramuscular fat and marbling were determined using ultrasonic scanning in Bali cattle. Two SNPs (g.5869T > C and g.5959C > T) in the *CAPN1* gene were significantly associated ( P<0.05 ) with the intramuscular fat percentage and marbling score. Marbling has been demonstrated to affect beef flavor, juiciness, tenderness, and visual qualities (Hwang et al., 2014; Silva and Cadavez, 2012). It also has a high heritability of 0.56. According to Park et al. (2018), a significant positive correlation (0.91) between tenderness and marbling supports the idea that marbling-based selection can significantly increase beef tenderness. Marbling is deposited within the endomysium, resulting in the weakening of the perimysial connective tissue and a more tender product (Nishimura, 2010). Prior studies demonstrated that several SNPs in the *CAPN1* gene were significantly associated ( P<0.05 ) with intramuscular fat content and marbling scores in Hanwoo and Nellore cattle (Cheong et al., 2008; Li et al., 2013). Previous research found that the SNP g.232G > C in the 5 ${}^{\prime }$ UTR of the *CAPN1* gene is significantly associated ( P<0.05 ) with the marbling score of Bali cattle (Dairoh et al., 2021). The findings confirm that genetic variants in *CAPN1* serve as possible markers that can be used in breeding programs to selectively improve marbling scores and intramuscular fat content in beef cattle, positively contributing to beef quality.

**Table 5:** Association of *CAPN1* gene SNPs with physical traits in Bali cattle.

SNP	Genotype ( n )	pH	WBSF	Cooking loss (%)	Color	WHC
g.5327C > T	CC (30)	$5.60\pm 0.11^{b}$	4.81±1.43	50.33±9.55	$3.00\pm 0.03^{b}$	29.12±1.95
	CT (11)	$5.68\pm 0.14^{a,b}$	4.94±1.18	52.2±9.17	$3.06\pm 0.27^{b}$	28.57±2.62
	TT (3)	$5.77\pm 0.08^{a}$	3.97±1.19	45.68±0.40	$3.33\pm 0.58^{a}$	30.90±1.77
g.5534C > T	CC (33)	5.62±0.12	4.76±1.42	49.87±9.23	3.00±0.03	29.20±1.93
	CT (10)	5.67±0.13	4.80±1.23	53.03±9.18	3.06±0.28	28.45±2.72
	TT (1)	5.69± nc	5.30± nc	45.45± nc	4.00± nc	31.81± nc
g.5807A > C	AA (31)	5.60±0.12	4.75±1.45	50.18±9.43	3.00±0.03	29.10±1.91
	AC (12)	5.69±0.13	4.83±1.19	51.70±8.91	3.05±0.26	28.86±2.69
	CC (1)	5.69± nc	5.30± nc	45.45± nc	4.00± nc	31.81± nc
g.5857G > A	GG (34)	5.64±0.12	4.80±1.35	51.34±9.68	3.04±0.23	29.09±2.13
	GA (10)	5.60±0.14	4.73±1.42	47.58±6.57	2.99±0.04	29.11±2.29
g.5869T > C	TT (3)	5.69±0.06	$6.05\pm 1.56^{a}$	50.40±11.18	$3.33\pm 0.58^{a}$	28.98±3.67
	TC (19)	5.64±0.15	$4.34\pm 0.90^{b}$	48.52±7.52	$3.03\pm 0.20^{b}$	29.42±2.29
	CC (22)	5.61±0.10	$5.00\pm 1.53^{a,b}$	52.19±10.20	$3.00\pm 0.02^{b}$	28.83±1.86
g.5959C > T	CC (3)	5.69±0.06	$6.05\pm 1.56^{a}$	50.40±11.18	$3.33\pm 0.58^{a}$	28.98±3.67
	CT (19)	5.64±0.15	$4.34\pm 0.90^{b}$	48.52±7.52	$3.03\pm 0.20^{b}$	29.42±2.29
	TT (22)	5.61±0.10	$5.00\pm 1.53^{a,b}$	52.19±10.20	$3.00\pm 0.02^{b}$	28.83±1.86

*CAPN1*: calpain 1; WBSF: Warner–Bratzler shear force; WHC: water-holding capacity; nc: not counted. ${}^{a,b}$ Means in the same column with different superscripts were significantly different ( P<0.05 ). Values ​​with the superscript a indicate different and higher values compared to those with the superscript b.

### 3.4 Association of *CAPN1* gene SNPs with physical traits

The associations of *CAPN1* gene polymorphisms with physical traits were analyzed in Bali cattle ( n=44 ). The evaluated traits were pH, WBSF, cooking loss, meat color, and WHC. The results are presented in Table 5. g.5327C > T in the *CAPN1* gene was significantly associated ( P<0.05 ) with pH and meat color in Bali cattle. The association study revealed that g.5869T > C and g.5959C > T were significantly associated ( P<0.05 ) with WBSF and meat color. Regarding the SNP g.5959C > T of the *CAPN1* gene, Bali cattle with the CT genotype had significantly lower WBSF than those with the TT and CC genotypes ( P<0.05 ). Tenderness was measured by shear force, with lower shear force values indicating greater tenderness. According to these findings, Bali cattle with the CT genotype of g.5959C > T were classified as tender, whereas those with the TT and CC genotypes were classified as harsh. Thus, the CT genotype is more desirable for high-quality meat. Tenderness is an essential meat quality attribute because variations in tenderness are suggested to influence consumers' decision to repurchase meat (Maltin et al., 2003). Several studies investigated the effect of genetic markers in the *CAPN1* gene on beef tenderness (Tizioto et al., 2013; Magalhães et al., 2019; Bedhane et al., 2019). Previous studies revealed that SNPs in the *CAPN1* gene are associated with tenderness (Curi et al., 2010; Sun et al., 2018). Therefore, these genetic markers might help select populations for beef breeding.

Prior studies demonstrated that the activity of proteolytic enzymes, including *CAPN1* (also known as μ -calpain), influenced tenderness. Calpain can degrade muscle proteins such as titin, nebulin, vinculin, and desmin, which play roles in meat tenderness (Bhat et al., 2018). Calpain has greater ability to degrade the titin protein, which has a molecular weight (MW) of 2400 kDa, than it does to degrade titin, which has a MW of 3700 kDa (Iwanowska et al., 2011). In addition, the tenderness of meat can be influenced by pH and WHC during post mortem examination. Strong evidence has revealed that low pH post mortem was associated with pale meat and poor WHC, resulting in poor meat quality and particularly low tenderness. Rapid pH decline can cause proteolytic denaturation or autolysis, reducing tenderness (Santos et al., 2016).

**Table 6:** Association of *CAPN1* gene SNPs with the fatty acid composition in Bali cattle.

	g.5327C > T			g.5534C > T			g.5807A > C			g.5857G > A		g.5869T > C			g.5959C > T		
Fatty acid	CC	CT	TT	CC	CT	TT	AA	AC	CC	GG	GA	CC	TC	TT	CC	CT	TT
composition	(29)	(11)	(3)	(32)	(10)	(1)	(30)	(12)	(1)	(34)	(9)	(22)	(18)	(3)	(3)	(18)	(22)
Fat content	3.33	2.80	3.74	3.36	2.72	3.77	3.33	2.90	3.77	3.18	3.39	3.33	3.04	3.47	3.47	3.04	3.33
C8:0	0.07	0.06	0.08	0.04	0.13	0.00	0.04	0.11	0.00	0.04	0.11	0.02	0.06	0.33	0.33 ${}^{a}$	0.06 ${}^{b}$	0.02 ${}^{b}$
C12:0	0.60	0.60	0.52	0.07	0.06	0.07	0.07	0.07	0.07	0.07	0.07	0.07	0.08	0.05	0.05	0.08	0.07
C13:0	21.09	20.96	23.21	0.03	0.03	0.03	0.02	0.03	0.03	0.03	0.03	0.02	0.03	0.02	0.06	0.09	0.10
C14:0	32.15	31.54	33.05	2.18	2.22	2.41	2.11	2.39	2.41	2.24	2.03	2.09	2.32	2.24	2.24	2.32	2.09
C14:1	0.42	0.38	0.45	0.28	0.27	0.05	0.30	0.24	0.05	0.30	0.19	0.33	0.25	0.04	0.04	0.25	0.33
C15:0	0.10	0.11	0.08	0.61	0.51	0.74	0.60	0.56	0.74	0.62	0.51	0.63	0.56	0.55	0.55	0.56	0.63
C16:0	0.09	0.09	0.08	21.33	20.70	22.23	21.15	21.27	22.23	21.06	21.74	20.50	21.94	22.00	22.00	21.94	20.50
C16:1	0.04	0.03	0.04	1.35	1.26	1.30	1.34	1.30	1.30	1.30	1.45	1.26	1.44	1.14	1.14	1.44	1.26
C17:0	0.06	0.03	0.05	2.06	1.88	1.73	2.07	1.90	1.73	1.90	2.43	1.92	2.09	2.19	2.19	2.03	1.92
C17:1	0.04	0.12	0.00	0.29	0.20	0.24	0.28	0.25	0.24	0.27	0.27	0.28	0.28	0.20	0.20	0.28	0.28
C18:0	0.02	0.14	0.03	32.24	31.19	34.19	32.15	31.59	34.19	31.79	32.99	31.77	32.13	33.56	33.56	32.13	31.77
C18:1n9t	2.07	1.78	1.89	3.13	2.54	3.46	2.98	3.03	3.46	3.08	2.71	3.07	2.94	2.86	2.86	2.94	3.07
C18:1n9c	0.31	0.26	0.30	11.77	13.40	13.77	11.74	13.20	13.77	12.08	12.63	12.31	11.52	15.40	15.40	11.52	12.31
C18:2n6c	1.34	2.14	1.45	1.87	1.56	1.91	1.90	1.53	1.91	1.73	2.05	1.71	1.97	1.39	1.39	1.97	1.71
C18:2n9t	11.64	12.08	15.06	0.14	0.04	0.02	0.13	0.08	0.02	0.12	0.07	0.15	0.09	0.02	0.02	0.09	0.15
C18:3n3	1.92	1.39	1.59	0.19	0.30	0.45	0.17	0.34	0.45	0.23	0.20	0.21 ${}^{b}$	0.19 ${}^{b}$	0.55 ${}^{a}$	0.55 ${}^{a}$	0.19 ${}^{b}$	0.21 ${}^{b}$
C18:3n6	0.01	0.05	0.02	0.01	0.00	0.03	0.01	0.00	0.03	0.01	0.01	0.01	0.01	0.01	0.01	0.01	0.01
C20:0	2.93	2.92	3.31	0.10	0.12	0.13	0.10	0.12	0.13	0.11	0.10	0.10	0.10	0.12	0.35	0.42	0.42
C20:1	0.18	0.37	0.16	0.35	0.24	0.08	0.37	0.21	0.08	0.31	0.38	0.33	0.36	0.04	0.04	0.36	0.33
C20:2	0.11	0.10	0.08	0.11	0.08	0.06	0.11	0.09	0.06	0.11	0.07	0.12	0.08	0.09	0.09	0.08	0.12
C20:3n3	0.11	0.11	0.13	0.10	0.12	0.19	0.11	0.11	0.19	0.11	0.10	0.11	0.11	0.10	0.10	0.11	0.11
C20:3n6	0.19	0.15	0.12	0.06	0.00	0.00	0.06	0.00	0.00	0.04	0.06	0.06	0.03	0.00	0.00	0.03	0.06
C20:4n6	0.16	0.34	0.04	0.17	0.20	0.19	0.18	0.17	0.19	0.18	0.17	0.17	0.21	0.10	0.10	0.21	0.17
C20:5n3	0.00	0.01	0.01	0.17	0.13	0.00	0.16	0.15	0.00	0.17	0.11	0.15	0.18	0.00	0.00	0.18	0.15
C21:0	0.36	0.15	0.39	0.42	0.40	0.47	0.42	0.39	0.47	0.42	0.39	0.42	0.42	0.35	0.35	0.42	0.42
C22:0	0.13	0.07	0.09	0.18	0.16	0.23	0.19	0.15	0.23	0.17	0.20	0.17	0.19	0.13	0.13	0.20	0.17
C23:0	0.00	0.02	0.01	0.09	0.09	0.11	0.09	0.09	0.11	0.10	0.07	0.10	0.09	0.06	0.06	0.09	0.10
C24:0	0.02	5.74	0.01	0.04	0.03	0.00	0.04	0.03	0.00	0.03	0.05	0.04	0.04	0.01	0.01	0.04	0.04
C24:1	0.28	1.50	0.29	0.01	6.32	0.00	0.02	5.26	0.00	1.87	0.02	0.01	3.52	0.00	0.00	3.52	0.01
SFA	16.88	18.07	20.83	59.51	54.32	62.38	59.16	56.05	62.38	57.72	60.81	57.95	58.33	61.67	61.67	60.93	57.95
MUFA	2.87	6.56	2.26	17.20	16.73	18.95	17.02	17.25	18.95	16.99	17.65	17.59	16.15	19.70	19.70	16.15	17.59
PUFA	19.75	24.63	23.10	2.82	3.83	2.85	2.83	3.64	2.85	3.12	2.83	2.69	3.64	2.25	2.25	3.64	2.69
UFA	57.88	59.56	62.14	20.02	20.82	21.80	19.85	21.13	21.80	20.17	20.48	20.28	19.88	21.95	21.95	19.88	20.28
T. fatty acid	78.70	72.79	85.23	79.39	75.89	84.17	78.88	77.74	84.17	78.15	80.72	78.07	78.86	82.17	82.17	78.86	78.07

*CAPN1*: calpain 1; SFA: saturated fatty acid; MUFA: monounsaturated fatty acid; UFA: unsaturated fatty acid; T. fatty acid: total fatty acid; nc: not counted. ${}^{a,b}$ Means in the same row with different superscripts were significantly different ( P<0.05 ). Values ​​with the superscript a indicate different and higher values compared to those with the superscript b.

### 3.5 Association of *CAPN1* gene SNPs with fatty acid content in Bali cattle

The associations of *CAPN1* gene SNPs with the fatty acid composition of Bali cattle were examined, as presented in Table 6. Linolenic acid (C18:3n3) content was significantly associated with the intronic SNP, g.5869T > C ( P<0.05 ). Specifically, the linolenic acid (C18:3n3) content was substantially greater in Bali cattle with the CC genotype than in cattle with the CT and TT genotypes. Linolenic acid (C18:3n3) is an omega-3 fatty acid and an essential fat that can only be obtained in food. Omega-3 fatty acids serve as substrates for the production of longer-chain omega-3 fatty acids, such as docosahexaenoic acid (C22:6 n-3) and eicosapentaenoic acid (C20:5 n-3), which are crucial for controlling blood pressure, inflammation, immune responses, brain development, and cognitive function (Sirot et al., 2008; Whitmore et al., 2019). Furthermore, omega-3 fatty acids have been demonstrated to contribute to preventing cardiovascular disease, diabetes, hypertension, allergies, cancer, renal disorders, and neural dysfunction and improving immune responses (Sinclair, 2019; Sokoła-Wysoczańska et al., 2018; Djuricic and Calder, 2021). Previous research found that SNPs in the exon 7 region of the *CAPN1* gene were associated with myristic, palmitic, linoleic, and linolenic acid levels in Yanbian cattle (Xin et al., 2011). A previous study also reported that several polymorphisms in the SCARB1 and SREBF1 genes were significantly associated ( P<0.05 ) with omega-3 fatty acid levels in Bali cattle (Dairoh et al., 2023).

Furthermore, the current study observed significant associations ( P<0.05 ) of the exon 10 SNP, g.5959C > T, with caprylic acid (C8:0) and linolenic acid (C18:3n3) levels. Caprylic acid (C8:0), a medium-chain fatty acid, has been proven to reduce body weight while improving internal fat accumulation and cholesterol metabolism (Rego-Costa et al., 2012). Previously, Roopashree et al. (2021) indicated that medium-chain saturated fatty acids are utilized to treat several conditions, including high weight, high cholesterol levels, cardiovascular diseases, and Alzheimer's disease, and used for their immunomodulatory effects. According to the present study, beef cattle with desired SNPs in the *CAPN1* gene might have high levels of fatty acids linked to selection. Furthermore, the newly discovered SNPs in these genes can be a great source of knowledge for enhancing meat quality, especially regarding fatty acids. More research in larger populations and diverse settings is necessary to validate the impact of the identified genetic variants.

**Table 7:** Average Ct, Δ Ct, and t test results for the *CAPN1* gene using the housekeeping gene β -actin in Bali cattle.

Genotype	Ct		Δ Ct	t test			
	*CAPN1*	β -actin		CC vs. CT	CC vs. TT		
CC	31.60±1.58	24.47±1.16	6.64±0.81	s	ns		
CT	31.91±1.50	21.93±2.59	9.97±1.31				
TT	31.47±1.53	24.14±2.40	7.34±1.06				

Ct: cycle threshold; Δ Ct: delta cycle threshold; *CAPN1*: calpain 1; s: significant; ns: not significant.

### 3.6 Total mRNA expression of the *CAPN1* gene in Bali cattle

*CAPN1* gene expression in liver tissue was classified according to the *CAPN1* g.5959C > T genotype. Tables 5 and 6 present the associations of g.5959C > T with WBSF and fatty acid content. The statistical results in Table 7 indicate that animals with the CT genotype had a higher mRNA expression than those with the CC genotype ( P=0.036 ), whereas no difference was observed between the CT and TT genotypes or between the CC and TT genotypes. The increase in *CAPN1* gene expression for the CT genotype indicated that muscle degradation was accelerated in Bali cattle, resulting in a lower shear force value (greater tenderness). Coria et al. (2020) found that greater shear force was associated with reduced *CAPN1* gene expression. Previous research revealed that the higher shear force of zebu meat was most likely attributable to enhanced *CAST* expression rather than decreased expression of protease-encoding genes (Giusti et al., 2013). The present study also found that the CT genotype was associated with greater mRNA expression and decreased accumulation of linolenic acid (C18:3n3) and caprylic acid (C8:0). These findings indicate that the *CAPN1* gene might influence meat quality and fatty acid content in Bali cattle. This finding could be used in future studies to identify the genetic basis for improved tenderization. Furthermore, the degree of *CAPN1* expression under shear force needs to be clarified in future research on diverse tissues and a greater number of Bali cattle.

## 4 Conclusions

In this study, six new SNPs in the *CAPN1* gene were found to be polymorphic in Bali cattle. SNPs g.5869T > C and g.5959C > T were significantly associated with the intramuscular fat percentage, marbling score, WBSF, meat color, and caprylic acid (C8:0) and linolenic acid (C18:3n3) content. Some beneficial features were observed in animals with the CT genotype of the SNP g.5959C > T. The CT genotype was linked to lower shear force (greater tenderness) and lower caprylic acid content (C8:0). The findings for the CT genotype differed significantly from those for the CC and TT genotypes. *CAPN1* gene polymorphisms could be used as markers for the intramuscular fat percentage, marbling score, tenderness, and fatty acid composition in Bali cattle.

## Data Availability

The datasets used in this study are available upon request from the author.
